# The function and impact of cryptocurrency and data technology in the context of financial technology: introduction to the issue

**DOI:** 10.1186/s40854-021-00301-w

**Published:** 2021-10-31

**Authors:** Lin Zhao

**Affiliations:** Southwestern University of Finance and Economics, Chengdu, China

With the rapid development of science and technology, financial technology (Fintech) has continuously made new breakthroughs, promoting the upgrading and innovation of financial model and reshaping the supply chain and value chain for financial industry. As an important product of Fintech, the cryptocurrency reduces the dependence of financial trading on financial intermediaries and contributes to the growth of digital economy. At the same time, certain key data processing technologies improve the efficiency of resource allocation and facilitate the reform and transformation of financial markets. The papers collected in this special issue aim at exploring new challenges that cryptocurrency brings to the financial market and discuss the role of some specific data processing technologies in the context of Fintech.

This volume is the29th issue of Financial Innovation (FIN), Volume 8, No. 5 (2021). In this issue, scholars from Canada, China, UK, Korea, Turkey, Russia have contributed their most up-to-date studies mainly on the new technologies used in the financial markets. The 15 papers explore state-of-the-art technologies including, but not limited to, the following areas:

- Financial Technology (3 papers)

Financial technology has been integrated into all aspects of our lives, promoting the continuous innovation and development of finance and business models.

The paper “Financial technology and the future of banking” presents an analytical framework that describes the business model of banks. It draws on the classical theory of banking and the literature on digital transformation. It provides an explanation for existing trends and, by extending the theory of the banking firm, it illustrates how financial intermediation will be impacted by innovative financial technology applications.

The paper “Insights into financial technology (FinTech): A bibliometric and visual study” conducts a comprehensive analysis based on bibliometrics and science mapping analysis for FinTech. Combining the analysis with the current financial environment, the challenges and future development opportunities are discussed.

- Data processing technology (2 papers)

Data processing technology can effectively reduce financial transaction costs and help accurately meet the needs of investors.

The paper “Basel III FRTB: data pooling innovation to lower capital charges” conducts a macro-level examination on the nature of FRTB modellability while also serving as a useful guide in navigating both the increasing computational complexities as well as data retention requirements within the modern data-focused regulatory climate with frameworks such as Basel III, Solvency II, and MiFID II among others.

The paper “Network DEA based on DEA-Ratio” applies DEA-Ratio (DEA-R) to evaluate two-stage DMUs instead of traditional DEA, and developed two novel DEA-R models, namely, range directional DEA-R (RDD-R) and (weighted) Tchebycheff norm DEA-R (TND-R). The Taiwanese non-life insurance companies are revisited using these proposed approaches and the results from the proposed methods are compared with those from some other methods.

- Cryptocurrency (10 papers)

Cryptocurrency has been an important research highlight in the study of financial technology recently. The paper "Are suspicious activity reporting requirements for cryptocurrency exchanges effective?" analyzes the impact of a newly emerging type of anti-money laundering regulation that obligates cryptocurrency exchanges to report suspicious transactions to financial authorities; the paper "Cryptocurrencies, gold, and WTI crude oil market efficiency: a dynamic analysis based on the adaptive market hypothesis" examines the evolving oil market efficiency by applying daily historical data to the three benchmark cryptocurrencies (Bitcoin, Ethereum, and Ripple), gold, and West Texas Intermediate (WTI) crude oil; the paper “Linearity extensions of the market model a case of the top 10 cryptocurrency prices during the pre-COVID-19 and COVID-19 periods" investigates the appropriateness of the linear specification of the market model for modeling and forecasting the cryptocurrency prices during the pre-COVID-19 and COVID-19 periods; the paper “Lottery-like preferences and the MAX effect in the cryptocurrency market” investigates the significance of extreme positive returns in the cross-sectional pricing of cryptocurrencies; the paper “High frequency multiscale relationships among major cryptocurrencies: Portfolio management implications” examines the high frequency multiscale relationships and nonlinear multiscale causality between Bitcoin, Ethereum, Monero, Dash, Ripple, and Litecoin; the paper “Central Bank Digital Currency, Loan Supply, and Bank Failure Risk: A Microeconomic Approach” uses a microeconomic banking model to investigate the effects of introducing an economy-wide, account-type CBDC on a bank’s loan supply and its failure risk.

Bitcoin, as a relatively mature cryptocurrency, has aroused broad discussion in the academia. The paper “Dynamic spillovers between the term structure of interest rates, bitcoin, and safe‑haven currencies” explores the connectedness between the US yield curve components (i.e., level, slope, and curvature), exchange rates, and the historical volatility of the exchange rates of the main safe-haven fiat currencies (Canada, Switzerland, EURO, Japan, and the UK) and the leading cryptocurrency, the Bitcoin; the paper “On the Factors of Bitcoin’s Value at Risk” investigates the factors of Bitcoin’s tail risk, quantified by Value at Risk (VaR); the paper “The time-varying causal relationship between the Bitcoin market and internet attention” examines the time-varying Granger causality between the global Bitcoin market and internet attention; the paper “Implied Volatility Estimation of Bitcoin Options and the Stylized Facts of Option Pricing” aims to provide insights into the volatility smile in Bitcoin options and estimate the implied volatility of Bitcoin options through numerical approximation techniques, specifically the Newton Raphson and Bisection methods.

